# Postnatal betamethasone treatment in extremely preterm infants and risk of neurodevelopmental impairment: a cohort study

**DOI:** 10.1136/archdischild-2024-327360

**Published:** 2024-12-18

**Authors:** Linn Löfberg, Fredrik Serenius, Lena Hellstrom-Westas, Elisabeth Olhager, David Ley, Aijaz Farooqi, Olof Stephansson, Thomas Abrahamsson

**Affiliations:** 1 Department of Biomedical and Clinical Sciences, Linköping University, Linköping, Sweden; 2 Crown Princess Victoria Children's Hospital, Linköping, Sweden; 3 Department of Women’s and Children’s Health, Uppsala University, Uppsala, Sweden; 4 Pediatrics, Institution of Clinical Sciences, Lund, Sweden; 5 Pediatrics, Department of Clinical Sciences, Umeå University, Umeå, Sweden; 6 Clinical Epidemiology Unit, Department of Medicine–Solna, Karolinska Institutet, Stockholm, Sweden

**Keywords:** Neurodevelopment, Neonatology

## Abstract

**Objective:**

To evaluate if postnatal treatment with betamethasone in extremely preterm infants was associated with neurodevelopmental impairment (NDI) at 6.5 years of age.

**Design:**

Prospective cohort study.

**Setting:**

Extremely Preterm Infants in Sweden Study (gestational age <27 weeks, born 2004–2007).

**Patients:**

428 children born extremely preterm were assessed at 6.5 years of age, 115 treated with betamethasone and 313 not treated.

**Main outcome measures:**

NDI at 6.5 years of age. Evaluation at 6.5 years included cognitive testing with the Wechsler Intelligence Scale for Children-Fourth Edition (WISC-IV), neurological examination and a medical record review.

**Exposure:**

Treatment with postnatal betamethasone.

**Main outcome:**

Moderate to severe NDI at 6.5 years of age, defined as a composite including cerebral palsy, and/or impairment in cognition, hearing and vision.

**Results:**

Moderate to severe NDI was more prevalent in children treated with postnatal betamethasone (49% treated vs 26% not treated, p<0.001). Betamethasone-treated children had worse cognitive development with mean WISC-IV score of 75 (SD 13.7) vs 87 (SD 14.0, p<0.001). The effect was dose dependent: 1.35 mg/kg vs 1.0 mg/kg (p=0.01) in betamethasone-treated children with moderate to severe versus no or mild NDI, respectively. The differences remained after adjustment for potential confounders with logistic regression (adjusted OR (aOR) 1.80, 95% CI 1.14 to 3.21). The difference in NDI also remained after propensity score matching, with crude OR 2.82 (95% CI 1.42 to 5.61, p=0.003) and aOR 2.17 (95% CI 1.07 to 4.69, p=0.04).

**Conclusion:**

Postnatal treatment with betamethasone is associated with increased risk of NDI at 6.5 years.

**WHAT IS ALREADY KNOWN ON THIS TOPIC**

- Postnatal use of corticosteroids has been debated for decades. Dexamethasone for treatment/prevention of bronchopulmonary dysplasia in extremely preterm infants has been studied earlier and is considered safe in low to moderate doses at a postmenstrual age of 8–14 days.

**WHAT THIS STUDY ADDS**

- Postnatal use of betamethasone is associated with worse neurodevelopment at 6.5 years of age. This is the first long-term follow-up after postnatal betamethasone use.

**HOW THIS STUDY MIGHT AFFECT RESEARCH, PRACTICE OR POLICY**

- Our results suggest that postnatal betamethasone should be used with caution in extremely preterm infants.

## Introduction

 Children born extremely preterm have high risk for neurodevelopmental impairments (NDI), including cognitive deficits, cerebral palsy (CP) and impaired hearing and vision.^1 2^ Postnatal complications such as sepsis, necrotising enterocolitis (NEC) and bronchopulmonary dysplasia (BPD) may disrupt normal brain development, and BPD plays a role for neurological deficits later in life.^3^

Antenatal corticosteroid treatment with betamethasone, to facilitate lung maturation and reduce the risk of intraventricular haemorrhage (IVH), has been well studied and is considered safe.^4^ The use of postnatal corticosteroids is more controversial, and their use was discouraged in 2002 by the American Academy of Pediatrics Committee on the Fetus and Newborn, after recognition of serious neurological side effects related to dexamethasone.^5 6^ Postnatal corticosteroids are known to reduce inflammation seen in BPD, facilitate weaning of ventilator-dependent infants and improve respiratory outcome at school age.^7^,^9^

Betamethasone has been proposed as an alternative to dexamethasone due to an advantageous risk profile with lower risk of periventricular leukomalacia (PVL) and a more potent effect on lung maturation.^10^,^12^ A few studies on short-term outcomes have been published so far, favouring its use.^13 14^ A recent study did not find impaired neurodevelopment among infants treated with betamethasone and/or hydrocortisone hemisuccinate at follow-up at 24 months of corrected age.^15^

In 2004–2007, when the Swedish study (Extremely Preterm Infants in Sweden Study, EXPRESS)^16^ enrolled patients, postnatal corticosteroid treatment was commonly used to facilitate weaning from the ventilator. A short course of intravenous betamethasone (a cumulative dose of 1.05 mg/kg over 7 days, with a higher dose in the first 3 days and then tapered) was administered to infants who were ventilator dependent after more than 14 days of postnatal age and showed no improvement in the pulmonary disease.

The aim of this study was to investigate if postnatal treatment with betamethasone in extremely preterm infants, to facilitate weaning from mechanical ventilation, was associated with an increased risk of NDI at 6.5 years of age.

## Materials and methods

### Study design and population

Data from the EXPRESS study, a prospective, observational cohort study, were used to investigate the association between postnatal betamethasone treatment and neurological impairment at 6.5 years of age. Data were collected for all infants born in Sweden at <27 gestational weeks from 1 April 2004 to 31 March 2007.^16^ Children were examined by a paediatrician and a psychologist and underwent an interview according to a structured protocol. In total, 441 children were assessed at 6.5 years of age, 59 of them by medical record review. Data on administered intravenous postnatal steroids, including total dose and postnatal age at the time for steroid treatment, were prospectively collected according to the EXPRESS study protocol.

### Assessment

Cognition was assessed by psychologists using the Swedish version of the Wechsler Intelligence Scale for Children-Fourth Edition (WISC-IV).^17^ The full-scale IQ (FSIQ) scores of children who had been born extremely preterm were related to the mean FSIQ score of a control group comprising children born at term.^1^ CP was defined according to Bax *et al*.^18^ The severity of CP was determined with clinical examination by a local paediatrician through the Gross Motor Function Classification System (GMFCS).^19^ Hearing impairment was defined as deafness or dependence on hearing aids, examined at an ear-nose-throat clinic. Visual acuity was assessed by ophthalmologists and classified according to modified WHO criteria.^20^ Small for gestational age was defined as birth weight <−2 SD for gestational age (GA) of birth, cystic PVL was defined according to de Vries *et al*,^21^ IVH was graded according to Papile *et al*,^22^ culture-proven sepsis was defined as positive blood cultures of relevant microbes, and retinopathy of prematurity (ROP) in accordance with the International Classification for Retinopathy of Prematurity.^23^ NEC was defined as Bell’s stages 2–3, in accordance with staging by Bell *et al*,^24^ severe BPD was defined as need for at least 30% oxygen at a time corresponding to 36 weeks of gestation.^25^

### Missing data

**Figure 1:**
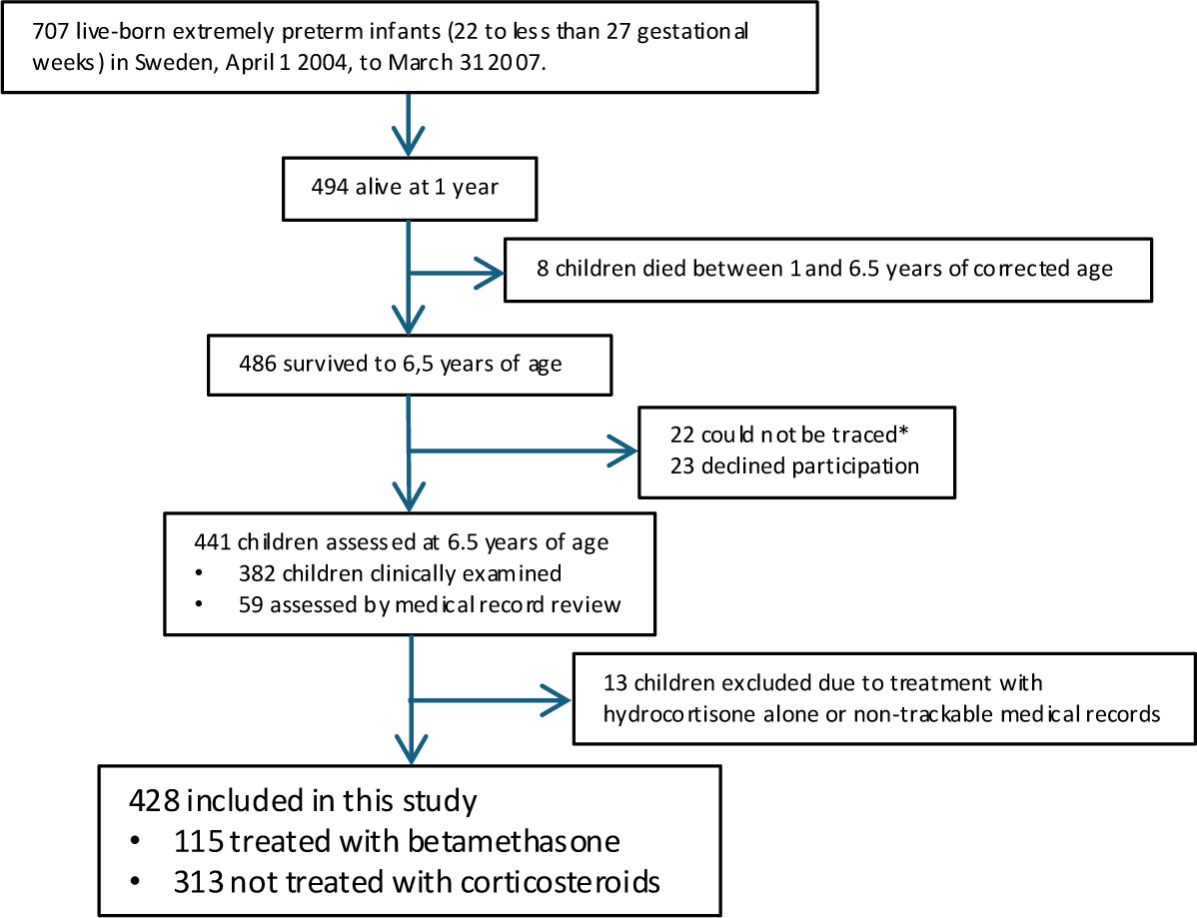
Flow chart of the study

Among 707 live-born infants, 484 survived to 6.5 years of whom 441 had follow-up (22 could not be traced and 23 declined participation, figure 1). Drop-out analysis has been presented earlier, and neonatal characteristics were similar in children participating and children not participating at the follow-up at 6.5 years of age.^1^ No data were missing for betamethasone, NDI or CP. No imputation was performed.

### Outcome

The primary outcome was moderate to severe NDI, which included a composite of cognitive impairment, CP and/or impairment of vision and hearing. Moderate NDI was defined as moderate cognitive disability based on FSIQ of −3 to −2 SD below mean or clinical/medical examination criteria, and/or CP severity based on GMFCS 2–3 and/or visual acuity of <20/63 but ≥20/400 in the better eye, and/or hearing loss corrected with hearing aid. Severe NDI was defined as FSIQ <−3 SD or severe cognitive disability based on clinical examination or medical chart review, and/or GMFCS ≥4, and/or blindness, and/or deafness (table 1). For further details, see Serenius *et al*.^1^ As secondary outcomes, the components of the composite outcome, diagnosis of CP and FSIQ were evaluated separately.

**Table 1:** The grading of neurodevelopmental impairment

	None	Mild	Moderate	Severe
Clinical examination/medical record review	None	Mild cognitive disability	Moderate cognitive disability	Severe cognitive disability
WISC-IV	>−1 SD	−2 to −1 SD	−3 to less than −2 SD	<−3 SD
Cerebral palsy	None	GMFCS 1	GMFCS 2–3	GMFCS≥4
Vision	None	Visual acuity <20/40 but ≥20/63 in the better eye	Visual acuity <20/63 but ≥20/400 in the better eye	Blindness
Hearing	None	None	Hearing loss corrected with hearing aid	Deafness

WISC-IV, Wechsler Intelligence Scale for Children IV; GMFCS, Gross Motor Function Classification System

GMFCS, Gross Motor Function Classification System; WISC-IV, Wechsler Intelligence Scale for Children-Fourth Edition.

### Statistical analyses

Independent samples t-tests were used to compare means between the groups for continuous variables, and Mann-Whitney U test was used to compare differences between groups in continuous or ordinal variables when the data were not normally distributed. The χ^2^ test (or Fisher’s exact test if the expected count was less than 5) was used to compare frequencies. Pearson’s correlation was used to investigate dose-dependent effects.

Confounding factors that were significantly associated both with betamethasone treatment and the specific outcome (moderate/severe NDI, moderate/severe cognitive impairment or CP at 6.5 years of age) were included in the logistic regression models, respectively.

**Figure 2:**
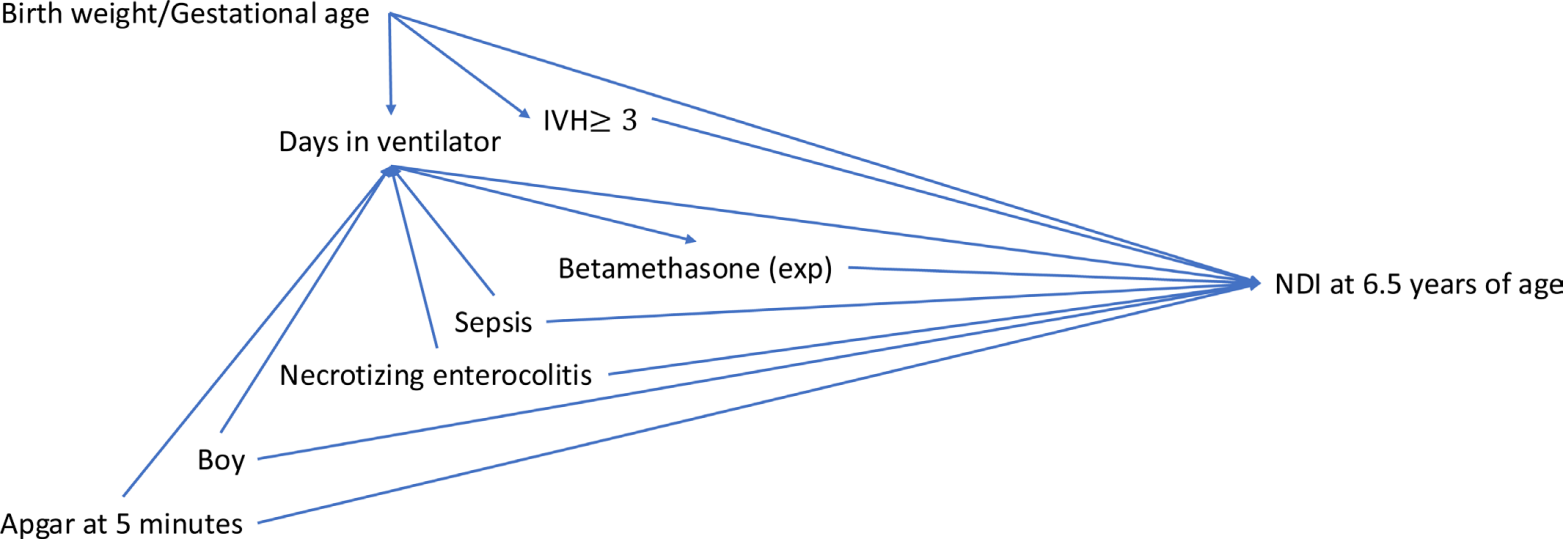
Directed acyclic graph of the study. IVH, intraventricular haemorrhage; NDI, neurodevelopmental impairment.

Propensity score was used to reduce the imbalance of the measured baseline characteristics between children treated with betamethasone and their matched controls. Directed acyclic graphs (DAG) were used to identify all potential confounding factors associated with NDI, occurring before exposure to betamethasone (figure 2).^26^ Chorioamnionitis, full-course antenatal steroids, GA, birth weight, sex, Apgar score ≥7 at 5 min, IVH≥3, culture-proven sepsis and NEC Bell’s stages 2–3 were identified based on previous studies.^227^,^30^ Children were matched 1:1, match tolerance was set to 0.2. This resulted in 208 matched children, 104 treated with betamethasone and 104 not treated. The propensity matched cohort was analysed regarding background characteristics, to control that it was well balanced (online supplemental table 3). The number of days on ventilator is closely related to the exposure, and considered a true confounder, and was not included in the propensity score. Conditional logistic regression was performed with the propensity scores and days on ventilator as predictors, and moderate to severe NDI as dependent variable.

The statistical analyses were carried out using the computer software program IBM SPSS for Windows V.25 (SPSS). All analyses were two sided and p values <0.05 were considered statistically significant. For propensity score matching and conditional logistic regression, STATA V.17 (StataCorp, USA) was used.

## Results

### Baseline characteristics

The study included 428 children, 115 treated with betamethasone in the neonatal period and 313 not treated (figure 1). Infants treated with betamethasone were born at a lower GA, had a lower birth weight and were more often boys compared with those not treated. Infants treated with postnatal corticosteroids were more often born in singleton births, had lower Apgar score at 5 min and had on average more days of ventilator treatment. They also had more complications such as culture-proven sepsis, persistent ductus arteriosus, BPD and ROP ≥stage 3. Median age for first dose of betamethasone was 18 days. There were no differences in educational level in parents when comparing treated and not treated (table 2).

**Table 2:** Background characteristics of extremely preterm children, who completed neurodevelopmental follow-up at 6.5 years, in relation to postnatal treatment with betamethasone (n=428)

	Postnatal betamethasone treatment		P value
	Yes	No	
**Birth characteristics**			
Age of mother, years, mean (SD)	31 (5.7)	31 (5.8)	0.64
Maternal smoking	10% (11/114)	13% (38/290)	0.34
Mother born in Nordic countries	84% (91/108)	80% (246/308)	0.32
Educational level of mother (years)			
≤9	11% (12/108)	9% (25/290)	
10–13	45% (49/108)	39% (114/290)	0.32*
≥14	43% (47/108)	52% (150/290)	
Educational level of father (years)			
≤9	13% (14/107)	12% (36/290)	
10–13	47% (50/107)	46% (134/290)	0.98*
≥14	40% (43/107)	41% (119/290)	
PPROM	19% (14/73)	22% (33/153)	0.68
Amnionitis	18% (20/110)	17% (48/290)	0.70
Maternal antibiotics	60% (68/114)	58% (68/292)	0.70
Parity 1st birth	55% (63/115)	60% (187/313)	0.36
Singleton birth	72% (83/115)	83% (259/313)	0.02
Full-course antenatal steroid	95% (109/115)	94% (276/295)	0.64
Caesarean section	55% (63/115)	57% (179/313)	0.66
Gestational age, week, mean (SD)	24.7 (1)	25.6 (1)	<0.001
Birth weight, g, mean (SD)	703 (139)	810 (171)	<0.001
Boy	61% (70/115)	50% (156/313)	0.04
Small for gestational age	17% (19/115)	16% (51/313)	0.96
Apgar score ≥7 at 5 min	52% (60/115)	75% (234/313)	<0.001
**Neonatal characteristics**			
Surfactant	80% (85/106)	51% (142/278)	<0.001
Days on ventilator, median (IQR)	22 (15–33)	5 (1–12)	<0.001
PDA pharmacological treatment	61% (70/115)	47% (148/313)	0.01
IVH ≥grade 3	12% (14/115)	9% (28/311)	0.33
Cumulative dose of betamethasone, mg/kg (median, IQR)	1.1 (0.88–2.0)	n/a	
Postnatal age, first steroid dose (median, IQR)	18 (12–25)	n/a	
**Events occurring after betamethasone exposure**			
Necrotising enterocolitis, Bell’s stages 2–3	5% (6/115)	5% (17/313)	0.93
Culture-proven sepsis	54% (62/115)	45% (140/313)	0.09
PDA surgery	38% (44/115)	22% (69/313)	0.001
ROP ≥stage 3	57% (66/115)	25% (79/310)	<0.001
Cystic periventricular leukomalacia	10% (11/115)	4% (14/313)	0.05
Severe BPD	34% (39/114)	18% (53/295)	<0.001

χ² test for dichotomised variables, t-test for continuous variables that were normally distributed, Mann-Whitney U test for comparison of medians.

* P value for overall differences between groups with available data was obtained with χ² test.

BPD, bronchopulmonary dysplasia; IVH, intraventricular haemorrhage; PDA, persistent ductus arteriosus; PPROM, preterm premature rupture of membranes; ROP, retinopathy of prematurity.

### Primary and secondary outcomes

Moderate to severe NDI was significantly more prevalent among children treated with betamethasone (49% vs 26%, p<0.001). The prevalence of CP was 12% in treated children and 8% in children not treated (p=0.18). The mean (SD) FSIQ score was 75 (13.7) in infants who received betamethasone and 87 (14.0) in non-treated infants (p<0.001) (online supplemental table 1). There was a weak correlation between FSIQ and total cumulative dose of betamethasone in milligram/kilogram (Pearson’s correlation −0.34 (95% CI −0.51 to −0.15, R^2^=0.117), p<0.001, n=91) (online supplemental figure 1). When comparing doses of betamethasone among treated children, those with moderate to severe NDI at 6.5 years of age had received higher doses of betamethasone compared with children with no or mild NDI, 1.35 mg/kg (IQR 1.0–2.8) vs 1.00 mg/kg (IQR 0.77–1.80) (p=0.02) (online supplemental table 2). Number needed to harm, that is, being diagnosed with moderate to severe NDI after betamethasone treatment, was 4.5 (95% CI 3.1 to 8.4).

Logistic regressions were performed with potential confounders associated with both betamethasone exposure and NDI at 6.5 years of age. Moderate to severe NDI was significantly associated with betamethasone exposure; crude OR for moderate to severe NDI was 2.63 (95% CI 1.69 to 4.10, p<0.001) and adjusted OR 1.80 (95% CI 1.14 to 3.21, p=0.01), after adjustment for GA, birth weight, male sex and number of days on ventilator. Betamethasone exposure was not significantly associated with CP (crude OR 1.60: 95% CI 0.80 to 3.19, adjusted OR 1.44: 95% CI 0.64 to 3.21, p=0.38; table 3).

**Table 3:** Postnatal betamethasone treatment and neurodevelopment at 6.5 years of age; results of logistic regression analysis of the whole study population (n=428)

	Crude OR	95% CI		Adj OR	95% CI		P value
		Lower	Upper		Lower	Upper	
Moderate to severe NDI	2.63	1.69	4.10	1.80*	1.08	3.00	0.03
Moderate or severe cognitive impairment	2.67	1.70	4.19	1.79†	1.06	3.02	0.03
Cerebral palsy (CP)	1.60	0.80	3.19	1.44‡	0.64	3.21	0.38

* Logistic regression with confounders gestational age, birth weight, boy and days on ventilator.

† Logistic regression with confounders gestational age, birth weight and days on ventilator.

‡ Logistic regression with confounders gestational age, birth weight, boy and days on ventilator.

Adj, adjusted; NDI, neurodevelopmental impairment.

A complementary analysis was performed after propensity score matching. The background characteristics of the propensity score matched cohort were well balanced regarding the factors included in calculation of the propensity score (online supplemental table 3). There was a difference in time spent on ventilator between children treated and children not treated (median 21 days (IQR 14–23) vs median 8 days (IQR 4–22), p<0.001). Conditional logistic regression, with moderate to severe NDI set as outcome, was performed after propensity score matching. Crude OR for NDI in the propensity matched cohort was 2.82 (95% CI 1.42 to 5.61, p=0.003). After adjustment for days on ventilator, adjusted OR was 2.17 (95% CI 1.07 to 4.69, p=0.04).

## Discussion

The data presented in this study indicate that postnatal betamethasone treatment used to wean extremely preterm infants off the ventilator is associated with an increased prevalence of moderate to severe NDI at 6.5 years. Betamethasone-treated children demonstrated lower IQ scores compared with those not treated. Among treated children, those with moderate to severe NDI at 6.5 years had received higher doses of betamethasone than those with no or mild NDI.

Long-term follow-up studies of infants after treatment with betamethasone have been lacking, mainly because dexamethasone previously has been the drug of choice worldwide. In the literature, we only found a small pilot study comparing betamethasone to dexamethasone, showing fewer adverse effects such poor weight gain and high blood glucose in the neonatal period.^13^ Previous studies comparing dexamethasone to betamethasone have shown similar potency at equivalent doses.^31^

The effects of dexamethasone have been extensively investigated. In a school age follow-up of neurological outcomes after a placebo-controlled randomised controlled trial (RCT) in 2004, early dexamethasone treatment was associated with reduced IQ scores,^32^ while another RCT with follow-up at 13–17 years of age did not show any differences between the dexamethasone and placebo groups.^33^ A meta-analysis by Doyle *et al*^34^ evaluated treatment with postnatal dexamethasone >7 days, in doses comparable to the betamethasone doses in this study, and found no associations between dexamethasone and CP. However, the investigators pointed out the lack of long-term follow-up studies.

In the present study, we found an increased prevalence of NDI after betamethasone treatment, although children were treated with lower betamethasone doses than the equivalent doses of dexamethasone previously considered safe.^34^ In contrast, a 15-year follow-up of a small RCT from 1990 evaluating long-term treatment with dexamethasone demonstrated a non-significant higher IQ score after a 42-day course of betamethasone (85 points) than 18 days (69 points) and placebo (73 points).^35^ This highlights the question of the balance of benefit and harm, suggesting that lower doses might lead to side effects but not the potential benefits.

The immature brain of the extremely preterm infant is undergoing rapid and complex developmental events, and adverse alterations in brain development that can be summarised as encephalopathy of prematurity.^36^ A study by Parikh *et al* reported reduced total brain volumes in extremely low birthweight infants treated with dexamethasone in the neonatal period,^37^ and a study by Cheong *et al* has reported these changes to persist into adulthood.^38^ PVL was not more common among children who developed NDI, but the fact that we only evaluated cystic PVL is a limitation in the study.^21^ The high rate of ROP in our cohort is also a marker of the vulnerability of these infants. The GA at birth was the strongest predictor for ROP in the EXPRESS study.^39^

The lack of a significant association between postnatal steroids and CP in the present study, noted in prior research,^5^ may stem from lower cumulative betamethasone doses in this study.

An important strength of this study is the 6.5 years of follow-up which is more predictive of future neurodevelopment than assessments at earlier ages. Furthermore, the EXPRESS trial was nationwide, population based and prospectively collected. The drop-out rate was low, and administered doses of betamethasone were eligible for included infants.

A major problem of the present study is confounding by indication for betamethasone treatment, which unidentified can introduce bias and lead to misinterpretation of the results. Potential confounders were identified by DAG and the analysis included both logistic regression analysis and propensity score matching. The results were similar in the models, which support the association between betamethasone treatment and NDI risk. We cannot rule out the risk of residual confounding. The most important potential confounder, however, was the duration of mechanical ventilation.

Number of days on ventilator is an indicator for vulnerability and other morbidities in the neonatal period. In our study, long mechanical ventilation was also a prerequisite for betamethasone treatment. Adjustment with logistic regression showed that the duration of mechanical ventilation remained significantly associated with moderate to severe NDI.

Another limitation of the present study is that the neonatal data originate from 2004 to 2007 and the interpretation in today’s setting must be made with caution. New ventilation strategies have emerged during the last 20 years, which might be more favourable to optimise ventilation and promote brain development. The field of nutrition has also expanded, optimising growth and brain development, and this is important to consider in further research and clinical practice.

## Conclusion

Postnatal treatment with betamethasone was associated with increased risk of moderate to severe NDI at 6.5 years, but not with CP. Children treated with betamethasone had a lower total score on WISC-IV. These findings advocate for a cautious approach to the use of postnatal betamethasone in neonatal care and highlight the need of RCTs that are well powered also for neurodevelopmental outcomes in older ages.

## Supplementary material

10.1136/archdischild-2024-327360 online supplemental figure 1

10.1136/archdischild-2024-327360 online supplemental table 1

## Data Availability

Data are available upon reasonable request. Data may be obtained from a third party and are not publicly available.
