# Supplementary figures and images for: Postnatal betamethasone treatment in extremely preterm infants and risk of neurodevelopmental impairment: a cohort study
Source: Arch Dis Child Fetal Neonatal Ed. 2024 Dec 18;110(4):e327360. doi: 10.1136/archdischild-2024-327360 (PMC12229056; doi:10.1136/archdischild-2024-327360)

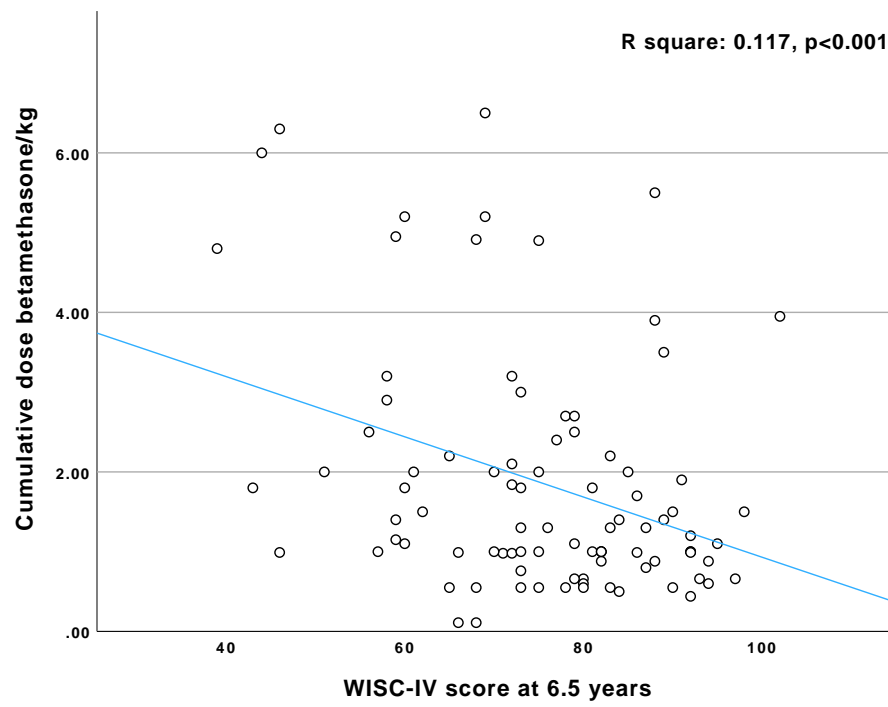

Supplement: online supplemental figure 1 [file fetalneonatal-110-4-s001.pdf]
