# Supplementary material for: Postnatal betamethasone treatment in extremely preterm infants and risk of neurodevelopmental impairment: a cohort study
Source: Arch Dis Child Fetal Neonatal Ed. 2024 Dec 18;110(4):e327360. doi: 10.1136/archdischild-2024-327360 (PMC12229056; doi:10.1136/archdischild-2024-327360)
Supplement: online supplemental table 1 [file fetalneonatal-110-4-s002.pdf]

Supplementary table 1. Postnatal Betamethasone treatment and outcome at 6.5 years follow up in the whole cohort (N=428).

	**Postnatal Betamethasone treatment**			
	**Yes**	**No**		
	**n=115**	**n=314**	P*	Odds Ratio (CI)
Moderate to Severe Neurodevelopment Impairment	49% (56/115)	27% (83/313)	<0.001	2.63 (1.69-4.10)
Cerebral Palsy (CP)	12% (14/115)	8% (25/313)	0.18	1.60 (0.80-3.19)
WISC-IV[a], points (sd), n/N	75 (13,7) (91/361)	87 (14,0) (270/361)	<0.001	11.1 (7.8-14.4)*

χ^2 test was employed for dichotomized variables. Fisher's exact test was used when the expected frequency for any cell was less than 5. Independent samples T-test was employed for continuous variables.

Independent samples T-test was employed for continuous variables

[a]Weschler Intelligence Scale for Children 4th edition

* Mean difference (CI)

Supplementary table 2. Background characteristics in the neonatal period for children with moderate to severe NDI at 6.5 years of age. N=428

	**Moderate to severe NDI**		
	Yes	No	
	% (n/N)	% (n/N)	P*
**Birth characteristics**			
Gestational age, wk, mean (sd)	25.1 (1.1)	25.5 (1)	<0.001
Birth weight in gram, mean (sd)	739 (160)	802 (170)	<0.001
Singleton birth	82% (114/139)	79% (228/289)	0.45
Boy	59% (82/139)	50% (144/289)	0.08
5-minute Apgar score ≥7	68% (94/139)	69% (200/289)	0.74
**Neonatal characteristics**			
Surfactant	61% (80/131)	58% (147/253)	0.58
Days on ventilator, median (IQR)	12 (3–27)	7 (2–18)	0.002
PDA[a] pharmacological treatment	50% (69/139)	52% (149/289)	0.71
IVH[b] ≥ grade 3	19% (27/139)	5% (15/287)	<0.001
Betamethasone treatment	40% (56/139)	20% (59/289)	<0.001
Cumulative dose betamethasone, mg/kg, median (IQR)*	1.35 (1.0–2.8)	1.00 (0.77–1.80)	0.02
**Events occuring after Betamethasone-exposure**			
Necrotizing enterocolitis	5% (7/139)	6% (16/289)	0.83
Culture-proven sepsis	48% (67/139)	47% (135/289)	0.77
PDA[a] surgical treatment	30% (42/139)	25% (71/289)	0.21
ROP[c] ≥ stage 3	48% (67/139)	28% (78/286)	<0.001
Cystic periventricular leukomalacia	8% (11/139)	5% (14/289)	0.21
Severe BPD[d]	31% (42/134)	18% (50/275)	0.003

χ^2 test was employed for dichotomized variables. Independent samples T-test was employed for continuous variables.

[a]Persistent Ductus Arteriousus

[b]Intraventricular hemorrhage

[c]Retinopathy of Prematurity

[d]Bronchopulmonary Dysplasia

* calculation based on 115 children treated with betamethasone

Supplementary table 3. Background characteristics for the propensity score matched cohort (N=208) in relation to postnatal treatment with betamethasone.

	Postnatal Betamethasone treatment		
	**Yes**	**No**	P
**Birth characteristics**			
Age of mother, years, mean (sd)	31.0 (5.6)	31.5 (5.6)	0.69
Maternal smoking	10% (10/103)	12% (11/93)	0.63
**Educational level mother**			
≤9 years	12% (12/98)	14% (13/95)	
10-13 years	44% (43/98)	37% (35/95)	0.61*
≥14 years	44% (43/98)	49% (47/95)	
**Educational level father**			
≤9 years	13% (13/97)	18% (17/94)	
10-13 years	43% (42/97)	38% (36/94)	0.62*
≥14 years	43% (42/97)	44 % (41/94)	
PPROM[a]	19% (12/64)	20% (11/56)	0.90
Amnionitis	19% (20/104)	21% (22/104)	0.73
Parity 1st birth	52% (54/104)	56% (58/104)	0.58
Singleton birth	73% (76/104)	88% (91/104)	0.01
Antenatal steroid full course	95% (99/104)	97% (101/104)	0.47
Caesarean section	56% (58/104)	49% (51/104)	0.33
Gestational age, wk, mean (sd)	24.8 (7)	24.9 (7)	0.17
Birth weight, g, mean (sd)	707 (144)	720 (157)	0.54
Boy	63% (65/104)	63% (65/104)	1.00
Small for gestational age	17% (18/104)	15% (16/104)	0.71
5-minute Apgar score≥7	51% (53/105)	64% (67/104)	0.05
**Neonatal characteristics**			
Surfactant	81% (78/96)	66% (64/97)	0.02
Days on ventilator, median (IQR)	21 (14-32)	8 (4-22)	<0.001
PDA[b] pharmacological treatment	59% (61/104)	47% (49/104)	0.10
IVH[c] ≥ grade 3	12% (12/104)	16% (17/104)	0.32
Cumulative dose betamethasone mg/kg (median, IQR)	1.1 (0.88-2.0)	n/a	
**Events occuring after Betamethasone-exposure**			
Necrotizing enterocolitis, Bells stage 2-3	6% (6/104)	8% (8/104)	0.58
Culture-proven sepsis	54% (56/104)	50% (52/104)	0.58
PDA[b] surgery	36% (37/105)	33% (34/104)	0.66
ROP[d] ≥ stage 3	57% (59/104)	30% (31/104)	<0.001
Cystic periventricular leukomalacia	11% (11/104)	6% (6/104)	0.21
Severe BPD[e]	33% (34/103)	19% (18/97)	0.02

χ^2 test for dichotomized variables, t-test for continuous variables that were normally distributed, and Mann-Whitney for data that were not normally distributed. Propensity score were calculated based on factors previously associated with neurodevelopment impairment occurring before treatment with postnatal

Betamethasone: amnionitis, full course antenatal steroids, gestational age, birth weight, sex, Apgar score at 5 minutes ≥7, IVH ≥3, culture-proven septicemia and NEC

*P value for overall differences between groups with avaliable data was obtained with χ^2 test.

[a]Preterm Premature Rupture of Membranes

[b]Persistent Ductus Arteriousus

[c]Intraventricular hemorrhage

[d]Retinopathy of Prematurity

[e]Bronchopulmonary Dysplasia
